# Modeling the trade-off between effective case management and imported malaria cases in different settings of *P. falciparum* malaria transmission intensity

**DOI:** 10.1186/1475-2875-9-S2-P7

**Published:** 2010-10-20

**Authors:** Valerie Crowell, Diggory Hardy, Nakul Chitnis, Nicolas Maire, Thomas Smith

**Affiliations:** 1 Department of Epidemiology and Public Health, Swiss Tropical and Public Health Institute, Basel, Switzerland

## Background

In many settings, *P. falciparum* transmission has been greatly reduced due to scaled-up malaria control. Imported cases will continue to be a source of malaria infection when vector control operations are stopped. We investigate the level of surveillance and case management that must be achieved in order to maintain reduced transmission levels, considering a varying rate of importation of cases and initial entomological inoculation rate (EIR).

## Methods

Simulations were carried out with a model ensemble based on a previously-published individual-based stochastic simulations of the natural history and epidemiology of *P. falciparum* malaria. As a reference we considered 1000 humans exposed to seasonal transmission based on a Tanzanian setting, with an initial EIR of two infectious bites per person per annum, and importation of infections at a rate of three per annum. Four annual rounds of indoor residual spraying were carried out at the beginning of the simulation and then stopped. Case management was simulated at varying coverages.

## Results

**Figure 1:**
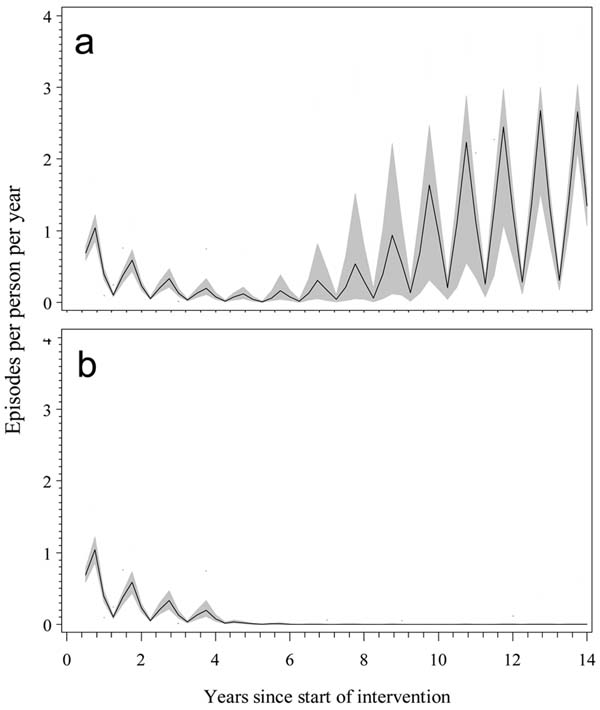
Predictions of clinical incidence with and without effective surveillance and case management, a: four annual IRS DDT spray rounds (each with 95% coverage); b: four annual IRS spray rounds followed by the introduction of case detection that eliminates 85% of secondary cases. Black lines: median; gray area: interquartile range; dashed lines: maxima and minima.

We present the results of a range of simulated scenarios. Figure 1a illustrates the reference situation with minimal case management coverage. The black lines give the median incidence of locally acquired cases from 10 simulation runs of each of 15 different models and parameterisations. The simulations suggest that, in this setting, malaria will resurge within several years. When case management eliminated 85% of secondary cases, clinical incidence remained negligible for the rest of the simulation period (Figure 1b).

## Conclusion

Initial results suggest that reductions in malaria transmission in many areas call for a strategy to deal with imported cases as a priority. In the absence of another transmission-blocking intervention, such as a vaccine, high coverage of case management will likely be critical to avoid resurgence of malaria.

